# Urethrectomy at the time of radical cystectomy for non-metastatic urothelial carcinoma of the bladder: a collaborative multicenter study

**DOI:** 10.1007/s00345-022-04025-z

**Published:** 2022-05-20

**Authors:** Ekaterina Laukhtina, Axelle Boehm, Benoit Peyronnet, Carlo Andrea Bravi, Jose Batista Da Costa, Francesco Soria, David D’Andrea, Pawel Rajwa, Fahad Quhal, Takafumi Yanagisawa, Frederik König, Hadi Mostafaei, Dmitry Enikeev, Alexandre Ingels, Gregory Verhoest, Frederiek D’Hondt, Alexandre Mottrie, Steven Joniau, Hendrik Van Poppel, Alexandre de la Taille, Karim Bensalah, Franck Bruyère, Shahrokh F. Shariat, Benjamin Pradere

**Affiliations:** 1 Department of Urology, Comprehensive Cancer Center, Medical University of Vienna, Währinger Gürtel 18-20, 1090 Vienna, Austria; 2 Institute for Urology and Reproductive Health, Sechenov University, Moscow, Russia; 3 Department of Urology, University Hospital of Tours, Tours, France; 4 Department of Urology, University Hospital of Rennes, Rennes, France; 5 Unit of Urology, Division of Oncology, URI, IRCCS Ospedale San Raffaele, Milan, Italy; 6 Department of Urology, Onze-Lieve-Vrouwziekenhuis Hospital, Aalst, Belgium; 7 ORSI Academy, Melle, Belgium; 8 Department of Urology, University Hospital Henri Mondor, AP-HP, UPEC, AP-HP, 51 Avenue du Maréchal de Lattre de Tassigny, 95010 Créteil Cedex, France; 9 Division of Urology, Department of Surgical Sciences, AOU Città della Salute e della Scienza di Torino, Torino School of Medicine, Turin, Italy; 10 Department of Urology, Medical University of Silesia, Zabrze, Poland; 11 Department of Urology, King Fahad Specialist Hospital, Dammam, Saudi Arabia; 12 Department of Urology, The Jikei University School of Medicine, Tokyo, Japan; 13 Department of Urology, University Medical Centre Hamburg-Eppendorf, Hamburg, Germany; 14 Research Center for Evidence Based Medicine, Tabriz University of Medical Sciences, Tabriz, Iran; 15 Department of Urology, University Hospitals Leuven, Leuven, Belgium; 16 Karl Landsteiner Institute of Urology and Andrology, Vienna, Austria; 17 Department of Urology, Weill Cornell Medical College, New York, NY USA; 18 Department of Urology, University of Texas Southwestern, Dallas, TX USA; 19 Department of Urology, Second Faculty of Medicine, Charles University, Prague, Czech Republic; 20 Hourani Center for Applied Scientific Research, Al-Ahliyya Amman University, Amman, Jordan; 21 Department of Urology, La Croix Du Sud Hospital, Quint Fonsegrives, France

**Keywords:** Bladder cancer, Radical cystectomy, Urethrectomy, Urethral recurrence

## Abstract

**Introduction:**

The optimal management of the urethra in patients planned for radical cystectomy (RC) remains unclear. We sought to evaluate the impact of urethrectomy on perioperative and oncological outcomes in patients treated with RC for non-metastatic urothelial carcinoma of the bladder (UCB).

**Materials and methods:**

We assessed the retrospective data from patients treated with RC for UCB of five European University Hospitals. Associations of urethrectomy with progression-free (PFS), cancer-free (CSS), and overall (OS) survivals were assessed in univariable and multivariable Cox regression models. We performed a subgroup analysis in patients at high risk for urethral recurrence (UR) (urethral invasion and/or bladder neck invasion and/or multifocality and/or prostatic urethra involvement).

**Results:**

A total of 887 non-metastatic UCB patients were included. Among them, 146 patients underwent urethrectomy at the time of RC. Urethrectomy was performed more often in patients with urethral invasion, T3/4 tumor stage, CIS, positive frozen section analysis of the urethra, and those who received neoadjuvant chemotherapy, underwent robotic RC, and/or received an ileal conduit urinary diversion (all *p* < 0.001). Estimated blood loss and the postoperative complication rate were comparable between patients who received an urethrectomy and those who did not. Urethrectomy during RC was not associated with PFS (HR 0.83, *p* = 0.17), CSS (HR 0.93, *p* = 0.67), or OS (HR 1.08, *p* = 0.58). In the subgroup of 276 patients at high risk for UR, urethrectomy at the time of RC decreased the risk of progression (HR 0.58, *p* = 0.04).

**Conclusion:**

In our study, urethrectomy at the time of RC seems to benefit only patients at high risk for UR. Adequate risk assessment of UCB patients’ history may allow for better clinical decision-making and patient counseling.

**Supplementary Information:**

The online version contains supplementary material available at 10.1007/s00345-022-04025-z.

## Introduction

The remnant urothelium remains at risk for disease recurrence after radical cystectomy (RC) for urothelial carcinoma of the bladder (UCB) [1]. Urethral recurrence (UR) is a relatively rare recurrence site after RC; it is mainly associated with risk factors such as tumor multifocality, papillary tumor pattern, carcinoma in situ (CIS), tumor at the bladder neck, and prostatic involvement [2, 3]. Prophylactic urethrectomy is nowadays rarely performed due to many reasons including increased use of orthotopic urinary diversion. Major guidelines do, indeed, not recommend routine urethrectomy; a negative urethral margin needs to be, however, confirmed before offering an orthotopic neobladder (ONB) [2, 4, 5]. Urethrectomy at the time of RC is only suggested in case of positive urethral frozen section analysis (FSA) and/or risk factors for UR [2, 4, 5].

These recommendations are mainly based on retrospective small cohorts and consensus opinions resulting in low-quality evidence. Moreover, the impact of urethrectomy (immediate or staged) on survival outcomes after RC remains unclear [6–8]. Consequently, management of the urethra during and after RC poses often a clinical dilemma. Therefore, we undertook a study to evaluate the impact of urethrectomy on perioperative and oncological outcomes in patients treated by RC for non-metastatic UCB.

## Materials and methods

### Study population

All procedures described in the present study were undertaken with the approval and oversight of the Institutional Review Board for the Protection of Human Subjects. This retrospective study included consecutive cohorts of patients who were treated with RC for non-metastatic UCB at five European medical centers between 1990 and 2020. Preoperative metastatic patients were excluded. The extent of lymphadenectomy and choice of urinary diversion as well as the decision to obtain FSA of the urethra and to perform urethrectomy was at the surgeon’s discretion. Neoadjuvant and adjuvant chemotherapy were administered at the clinicians’ discretion based on tumor stage and overall health status (including cisplatin-eligibility).

### Pathological review

All surgical specimens were processed according to standard pathological procedures. Pathological stage was established according to the 2011 American Joint Committee on Cancer TNM staging system. Genitourinary pathologists assigned tumor grade according to the 2004 WHO grading system. The presence of concomitant carcinoma in situ (CIS) was defined as the presence of CIS in conjunction with another tumor other than CIS [9]. Positive soft tissue surgical margin was defined as the presence of tumor at inked areas of soft tissue on the RC specimen [10].

### Follow-up

Clinical and radiological follow-up was performed in accordance with institutional protocols and current guidelines. Routine follow-up usually included physical examination, radiological imaging, and urine cytology. UR was defined as recurrence at the urethra or the region of the removed urethra. We evaluated rate of UR and the time to their occurrence. Tumor progression was defined as the occurrence of locoregional recurrence or distant metastasis on radiological imaging. Progression-free survival (PFS) time was calculated from the date of RC to tumor progression or last follow-up. Cause of death was abstracted from medical charts and/or from death certificates [11]. Overall survival (OS) time was calculated from the date of RC to death or last follow-up. Cancer-specific survival (CSS) time was calculated from the date of RC to death from disease or last follow-up.

### Statistical analysis

Report of categorical variables included frequencies and proportions. Continuous variables were reported as medians and interquartile ranges (IQR). With respect to urethrectomy, group comparisons were performed using the Wilcoxon rank sum, Pearson’s Chi-squared, and Fisher’s exact tests and subsequent significance testing, as appropriate. Association between urethrectomy or urethral FSA performance with PFS, CSS, and OS was assessed in univariable and multivariable Cox regression models. Analyses were also performed in a subgroup of patients at high risk for UR: patients with urethral invasion and/or bladder neck invasion and/or multifocality and/or prostatic urethra involvement. Criteria of high risk for UR have been chosen based on major guidelines and a recent meta-analysis [2, 3]. The risk of survival was expressed as hazard ratios (HR) and 95% confidence interval (95% CI). Kaplan–Meier survival curves were used to depict the association between urethrectomy or urethral FSA performance and survival. The log-rank test was used to determine the statistical difference between urethrectomy/non-urethrectomy and FSA/non-FSA groups with respect to progression or death. All reported *p* values were two-sided, and statistical significance was set at 0.05. All statistical analyses were performed using R Version 4.0.4.

## Results

### Clinicopathologic features

A total of 887 non-metastatic UCB patients were included in the analysis. The median age of the entire cohort was 67 years (IQR 60–74). Patient characteristics are shown in Table 1. More than half of the patients received an ileal conduit for urinary diversion (57%). Neoadjuvant chemotherapy was administered to 104 patients (12%), and adjuvant chemotherapy to 132 patients (15%).

**Table 1:** Association of urethrectomy performance with clinicopathologic characteristics in 887 patients treated with radical cystectomy for urothelial carcinoma of the bladder

Characteristic	Overall	Stratified by urethrectomy		
	*N* = 887	No, *N* = 741	Yes, *N* = 146	*p* value
Age (years)	67 (60, 74)	67 (60, 74)	67 (61, 73)	0.8
Gender				0.12
Male	781 (88%)	658 (89%)	123 (84%)	
Female	106 (12%)	83 (11%)	23 (16%)	
BMI	26.3 (23.8, 29.4)	26.3 (23.9, 29.4)	26.5 (23.2, 28.9)	0.8
ASA				0.2
1	121 (15%)	108 (16%)	13 (9.3%)	
2	429 (52%)	354 (52%)	75 (54%)	
3	259 (32%)	210 (31%)	49 (35%)	
4	13 (1.6%)	10 (1.5%)	3 (2.1%)	
History of NMIBC	502 (62%)	468 (63%)	34 (46%)	0.003
Pathology before RC				< 0.001
pTa	31 (3.6%)	26 (3.6%)	5 (3.5%)	
pT1	170 (20%)	139 (19%)	31 (22%)	
pT2	596 (69%)	520 (72%)	76 (53%)	
pT3/4	38 (4.4%)	21 (2.9%)	17 (12%)	
CIS	28 (3.2%)	13 (1.8%)	15 (10%)	
Urethral invasion before RC	186 (23%)	156 (22%)	30 (43%)	< 0.001
NAC	104 (12%)	69 (9.4%)	35 (24%)	< 0.001
Approach				< 0.001
Open	405 (46%)	355 (48%)	50 (34%)	
Laparoscopic	335 (38%)	315 (43%)	20 (14%)	
Robot	142 (16%)	67 (9.1%)	75 (52%)	
Urinary diversion				< 0.001
Ileal conduit	515 (58%)	377 (51%)	138 (95%)	
Orthotopic	320 (36%)	320 (43%)	0 (0%)	
Ureterostomy	46 (5.2%)	39 (5.3%)	7 (4.8%)	
FSA urethra	419 (54%)	401 (57%)	18 (25%)	< 0.001
Positive FSA urethra	24 (5.7%)	11 (2.7%)	13 (72%)	< 0.001
EBL (ml)	900 (500, 1,500)	900 (600, 1,500)	800 (350, 1,500)	0.05
Operative time (min)	330 (270, 380)	320 (260, 375)	350 (305, 411)	< 0.001
Final pathology stage				> 0.9
pT0/pTa/pTis/pT1	269 (31%)	223 (31%)	46 (32%)	
pT2	141 (16%)	117 (16%)	24 (17%)	
pT3/pT4	456 (53%)	383 (53%)	73 (51%)	
pN				0.2
N0	579 (67%)	491 (67%)	88 (67%)	
N +	206 (24%)	170 (23%)	36 (27%)	
Nx	85 (9.8%)	77 (10%)	8 (6.1%)	
Positive soft tissue surgical margin	123 (14%)	104 (14%)	19 (13%)	0.8
Histology				0.5
Urothelial	779 (91%)	662 (90%)	117 (92%)	
Variant histology	81 (9.4%)	71 (9.7%)	10 (7.9%)	
CIS on final specimen	323 (38%)	273 (39%)	50 (34%)	0.3
Adjuvant chemotherapy	132 (15%)	108 (15%)	24 (17%)	0.6
Complications according to Clavien–Dindo				0.7
Minor (grade 1 and 2)	450 (69%)	410 (69%)	40 (71%)	
Major (grade 3, 4, and 5)	98 (31%)	182 (31%)	16 (29%)	

Median (IQR); *n* (%)

Wilcoxon rank sum test; Pearson’s Chi-squared test; Fisher’s exact test

In our cohort, 146 patients underwent total urethrectomy at the time of RC. Patients who underwent urethrectomy, had more urethral invasion (*p* < 0.001), were more likely to have received neoadjuvant chemotherapy (*p* < 0.001), underwent more often robotic RC (*p* < 0.001), and were more likely to be clinically non-organ confined tumor stage before RC and have CIS (*p* < 0.001) or positive FSA of the urethra (*p* < 0.001). Two patients underwent staged urethrectomy (within two months after RC) due to the presence of a urethral margin on definitive RC pathology. Six patients (0.6%) underwent delayed urethrectomy due to UR.

FSA of the urethra was performed in 419 patients (54%) and 24 of patients who underwent FSA (5.7%) had positive results. Of those patients with positive urethral FSA, 13 (72%) underwent immediate urethrectomy and only three (12.5%) had a positive invasion on final specimen. Among patients with negative FSA of the urethra, four patients (10%) underwent urethrectomy; only one of them had a positive final margin. The non-urethrectomy group was more likely to have FSA of the urethra performed (57 versus 25%; *p* < 0.001).

The estimated blood loos (EBL) as well as postoperative complication rate (Supplementary Table 1) were comparable between urethrectomy and non-urethrectomy groups. Operative time was significantly longer for the urethrectomy group with a median of 320 versus 350 min, respectively (*p* < 0.001).

### Survival outcomes

Median follow-up of patients alive was 32 months (IQR 12–66). During the follow-up period, 362 patients died, 244 of them due to cancer; 371 patients experienced progression. Median time to progression was 38 months (IQR 9–67). Patients in the urethrectomy and non-urethrectomy groups had comparable tumor stages on the final RC pathology. The number of invaded nodes did not differ between the two groups (*p* = 0.2). A positive soft tissue surgical margin was identified in 123 (14%) patients: 104 (14%) in the non-urethrectomy group and 19 (13%) in the urethrectomy group. Eleven patients (1.2%) in the cohort had UR within the follow-up time. The median time from surgery to UR was 15 months, six patients with UR died due to UCB with a median time to death of 17 months. Rate of UR was 1.6% for orthotopic urinary diversion and 2.7% for transcutaneous diversion. Six patients who experienced UR (55%) underwent salvage urethrectomy.

Overall, the 3-year estimates for PFS, CSS, and OS were 53% (95% CI 49–57%), 68% (95% CI 64–72%), and 59% (95% CI 55–62%), respectively. In patients who underwent urethrectomy versus non-urethrectomy group, the 3-year PFS, CSS, and OS were 58% (95% CI 50–68%) versus 51% (95% CI 47–57%), 71% (95% CI 63–80%) versus 67% (95% CI 63–72%), and 60% (95% CI 52–69%) versus 58% (95% CI 54–63%), respectively. Urethrectomy at the time of RC was neither associated with PFS (HR 0.83, 95% CI 0.63–1.08, *p* = 0.17), CSS (HR 0.93, 95% CI 0.66–1.30, *p* = 0.67), nor OS (HR 1.08, 95% CI 0.83–1.40, *p* = 0.58) (Supplementary Fig. 1).

**Fig. 1:**
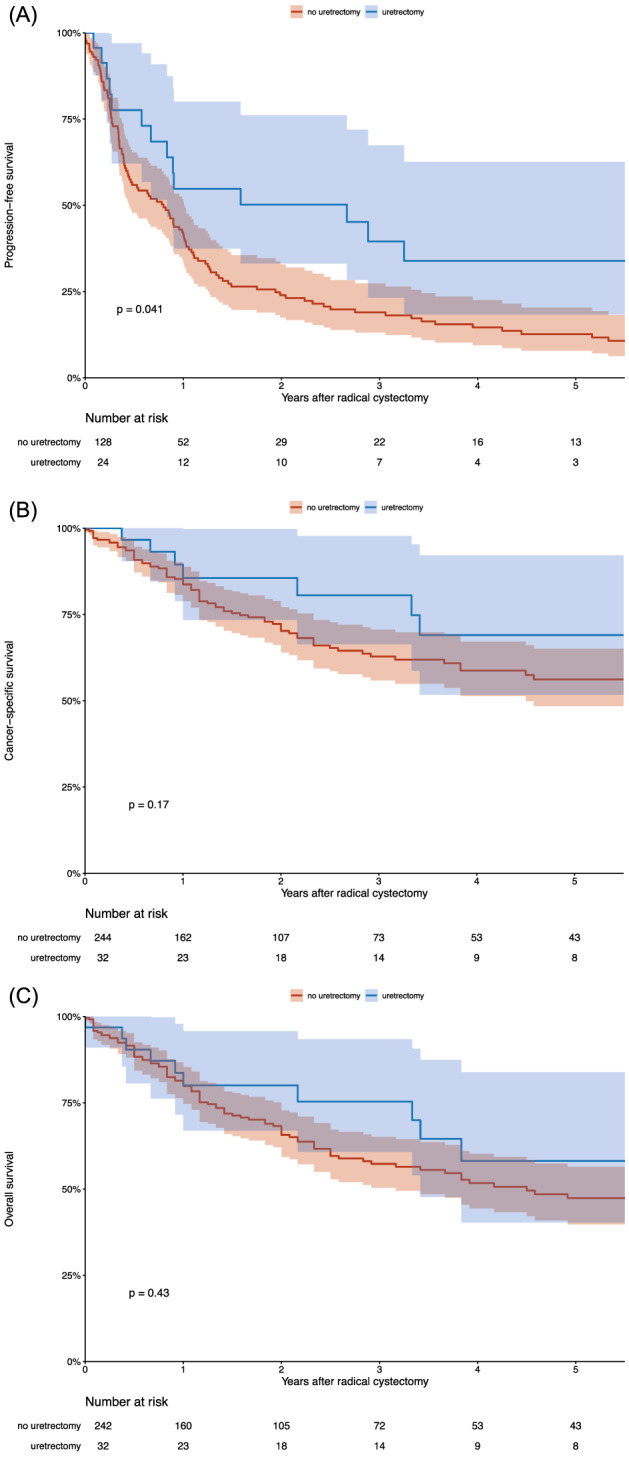
Kaplan–Meier analysis for **A** progression-free survival (PFS), **B** cancer-specific survival (CSS), and **C** overall survival (OS) in 276 high-risk patients treated with radical cystectomy for urothelial carcinoma of the bladder, stratified according to urethrectomy performance

In subgroup of patients at high risk for UR (*n* = 276) (Fig. 1), urethrectomy at the time of RC was associated with improved PFS (HR 0.58, 95% CI 0.34–0.99, *p* = 0.04), but not with CSS (HR 0.58, 95% CI 0.27–1.27, *p* = 0.17) and OS (HR 0.78, 95% CI 0.43–1.43, *p* = 0.43). On multivariable analysis, urethrectomy was not associated with PFS in patients of high risk for UR treated with RC for UCB (Table 2).

**Table 2:** Multivariable Cox regression models for the prediction of progression-free survival, cancer-specific survival, and overall survival in 276 high-risk patients treated with radical cystectomy for urothelial carcinoma of the bladder

Characteristic	Progression-free survival			Cancer-specific survival			Overall survival		
	HR	95% CI	*p* value	HR	95% CI	*p* value	HR	95% CI	*p* value
Urethrectomy	0.48	0.26, 0.87	0.02	0.57	0.26, 1.27	0.2	0.70	0.36, 1.35	0.3
Urethral FSA performance	0.76	0.52, 1.10	0.15	0.50	0.32, 0.79	0.003	0.50	0.33, 0.74	< 0.001
Pathology stage									
pT0/pTa/pTis/pT1	Ref	Ref		Ref	Ref		Ref	Ref	
pT2	2.88	1.13, 7.33	0.03	3.34	1.26, 8.91	0.02	2.31	1.07, 4.98	0.03
pT3/pT4	4.01	1.75, 9.15	< 0.001	5.50	2.27, 13.3	< 0.001	3.46	1.77, 6.75	< 0.001
Lymph node involvement	1.00	0.68, 1.48	> 0.9	1.59	0.97, 2.61	0.07	1.35	0.84, 2.16	0.2
Positive soft tissue surgical margin	1.71	1.12, 2.62	0.01	1.41	0.82, 2.42	0.2	1.55	0.95, 2.53	0.08
NAC	1.91	0.82, 4.41	0.13	1.22	0.48, 3.09	0.7	1.10	0.47, 2.57	0.8

*CIS *carcinoma in situ, *FSA *frozen section analysis, *HR *hazard ratio, *CI* confidence interval, *NAC* neoadjuvant chemotherapy

FSA of the urethra at the time of RC was associated with OS (HR 0.68, 95% CI 0.54–0.85, *p* < 0.001), but not with PFS (HR 0.82, 95% CI 0.66–1.02, *p* = 0.0772) and CSS (HR 0.77, 95% CI 0.59–1.01, *p* = 0.06). On multivariable analysis, urethral FSA performance remained significantly associated with OS (HR 0.68, 95% CI 0.54–0.86, *p = *0.001) (Supplementary Table 2). In patients with negative FSA who did not undergo urethrectomy, the 3-year PFS, CSS, and OS were 54% (95% CI 48–61%), 71% (95% CI 66–77%), and 64% (95% CI 59–69%), respectively. In the subgroup of patients at high risk for UR, FSA of the urethra at the time of RC was associated with both CSS (HR 0.56, 95% CI 0.36–0.86, *p* = 0.01) and OS (HR 0.53, 95% CI 0.36–0.78, *p* = 0.001). On multivariable analysis, urethral FSA performance remained significantly associated with both OS (HR 0.50, 95% CI 0.33–0.74, *p* < 0.001) and CSS (HR 0.50, 95% CI 0.32–0.79, *p* = 0.003) (Table 2).

## Discussion

We performed the largest collaborative multicenter study investigating the impact of urethrectomy at the time of RC on perioperative and oncological outcomes in patients treated with RC for non-metastatic UCB. In our study, urethrectomy did not seem to improve survival outcomes (PFS, CSS, OS) in every patient treated with RC for UCB. However, patients at high risk for UR were most likely to benefit from urethrectomy at the time of RC. In terms of perioperative outcomes, we observed comparable EBL as well as postoperative complication rate for the urethrectomy versus non-urethrectomy groups, while urethrectomy led to longer operative times.

Immediate urethrectomy does not seem to improve survival in all patients treated with RC for UCB. Our results are in line with previously published data [6, 8]. In a large study based on the SEER database including 195 men who underwent either immediate/staged (within 6 weeks after RC, 103 patients) or delayed (more than 6 weeks after RC, 92 patients) urethrectomy, Nelles et al. reported that urethrectomy did not appear to yield a significant disease-specific survival benefit [8]. Moreover, the authors observed that the timing of urethrectomy did not appear to impact survival. Similarly, Spiess et al. did not find differences in surgical morbidity or survival outcomes for patients who underwent immediate (57 patients) and staged (19 patients) urethrectomy [6]. Unfortunately, due to the limited number of patients who underwent staged or delayed urethrectomy in our cohort (two and six patients, respectively), it was not feasible to provide information and compare survival outcomes based on timing of urethrectomy.

Our analyses indicated improved PFS after urethrectomy at the time of RC in patients at high risk for UR. Similarly, Hakozaki et al. reported the survival benefit of prophylactic urethrectomy in patients at high UR risk (multiple tumors and/or concomitant CIS) [7]. Interestingly, that survival benefit was also found in patients who did not receive neoadjuvant chemotherapy (NAC) [7]. It can be hypothesized that patients at high risk for UR are more likely to benefit from NAC for downstaging and improving oncological outcomes, especially in those planned to spare urethra (e.g., ONB diversion). Unfortunately, due to a limited number of patients, we were not able to perform such subgroup analysis. Previous meta-analyses reported that male patients treated with non-ONB diversion, prostatic involvement, tumor multifocality, concomitant CIS, and positive urethral margins are at increased risk for UR [3, 12]. In addition, the AUA guidelines suggested papillary pattern and bladder neck involvement as risk factors for UR after RC [2]. The stratification based on risk factors should help in the decision-making to select those patients who are most likely benefit from immediate urethrectomy during RC.

Despite the possible survival benefit, urethrectomy might be associated with increased surgical morbidity. However, our analyses indicated comparable perioperative complication rates as well as EBL at the urethrectomy and the non-urethrectomy groups. It has been reported that urethrectomy complications are mainly genital hematoma [13]. Taking into account the surgical approach, the prepubic approach was shown to be associated with a lower risk of severe complications with shorter operative time and hospital length of stay compared to a perineal approach [14, 15]. Unfortunately, we could not provide data on the surgical approaches due to the retrospective nature of the study. However, we confirmed that urethrectomy at the time of RC led to longer operating times. This leads to longer analgesic consumption, delayed mobilization, and convalescence that might be harmful in elderly UCB patients with comorbidities. That might also increase the risk of deep venous thrombosis [13]. Hence, it supports the hypothesis that routine urethrectomy should be avoided in every patient treated with RC unless a high suspicion for urethral invasion is estimated. Accurate risk risk-adjusted patient classification is an unmet need of assessing UR risk factors in UCB patients.

We found that FSA of the urethra during RC improved survival outcomes (CSS and OS) in UCB patients, especially in those at high risk for UR. Although the accuracy and prognostic benefit of FSA during RC still remain controversial [16], high diagnostic performance for FSA of urethral margin during RC with a pooled sensitivity of 83% and specificity of 95% has been recently reported [17]. Intraoperative FSA is recommended to verify a negative urethral margin before offering ONB [2, 5]. In case of positive results, it can lead to immediate urethrectomy at the time of RC. While even in cases of risk factors, if the FSA of the urethra is negative, an ONB diversion can be safely performed [18]. Positive final pathological margin could lead to a staged urethrectomy without compromising patient survival [19].

The main strength of the present study is that, to our knowledge, this is the largest series investigating the impact of urethrectomy at the time of RC on oncological and perioperative outcomes in patients treated with RC for non-metastatic UCB. However, our study is not devoid of limitations. The main limitation of the study was its retrospective and multicenter design, which may result in a lack of pathologic and surgical approaches that could confound the results. Further, data on the urethrectomy approach, which might also alter outcomes, were, unfortunately, unavailable. Although the evaluation of local recurrence versus distant metastasis would have been interesting to investigate, the retrospective design of our study did not allow us to differentiate the type of recurrence. The small number of patients who underwent staged or delayed urethrectomy did not allow us to compare survival outcomes with regard to the timing of urethrectomy. Further well-designed studies should be conducted to establish selection criteria for urethrectomy and the extent of urethrectomy at the time of RC.

## Conclusion

In our study, urethrectomy at the time of RC did not improve survival outcomes for every patient treated with RC for UCB. However, patients at high risk for UR are most likely to benefit from urethrectomy at the time of RC. FSA of the urethra during RC may improve outcomes in UCB patients, especially in those at high risk for UR. The decision to perform immediate urethrectomy should take into consideration risk factors, pathologic findings, and patient comorbidities.

## Supplementary Information

Below is the link to the electronic supplementary material.

Supplementary file1 (DOCX 237 KB)
