# Supplementary material for: Urethrectomy at the time of radical cystectomy for non-metastatic urothelial carcinoma of the bladder: a collaborative multicenter study
Source: World J Urol. 2022 May 20;40(7):1689–96. doi: 10.1007/s00345-022-04025-z (PMC9236994; doi:10.1007/s00345-022-04025-z)
Supplement: Supplementary file 1 — Supplementary file1 (DOCX 237 KB) [file 345_2022_4025_MOESM1_ESM.docx]

**Supplementary Table 1. Association of urethrectomy performance with complication rate (according to Clavien-Dindo) in 887 patients treated with radical cystectomy for urothelial carcinoma of the bladder.**

|  | Overall | Stratified by urethrectomy | | |
| --- | --- | --- | --- | --- |
| Characteristic | N = 857 | No (N = 783) | Yes (N = 74) | p-value |
|  |  |  |  | >0.9 |
| Grade 1 | 186 (29%) | 169 (29%) | 17 (30%) |  |
| Grade 2 | 253 (40%) | 230 (40%) | 23 (41%) |  |
| Grade 3A | 26 (4.1%) | 24 (4.1%) | 2 (3.6%) |  |
| Grade 3B | 88 (14%) | 81 (14%) | 7 (12%) |  |
| Grade 4A | 42 (6.6%) | 39 (6.7%) | 3 (5.4%) |  |
| Grade 4B | 10 (1.6%) | 10 (1.7%) | 0 (0%) |  |
| Grade 5 | 32 (5.0%) | 28 (4.8%) | 4 (7.1%) |  |
| n (%) | | | | |
| Fisher's exact test | | | | |

**Supplementary Table 2. Multivariable Cox regression models for the prediction of progression-free survival, cancer-specific survival, and overall survival in 887 patients treated with radical cystectomy for urothelial carcinoma of the bladder.**

|  | Progression-free survival | | | Cancer-specific survival | | | Overall survival | | |
| --- | --- | --- | --- | --- | --- | --- | --- | --- | --- |
| Characteristic | HR | 95% CI | p-value | HR | 95% CI | p-value | HR | 95% CI | p-value |
| Urethrectomy | 0.83 | 0.56, 1.21 | 0.3 | 0.75 | 0.45, 1.25 | 0.3 | 0.79 | 0.52, 1.19 | 0.3 |
| Urethral FSA performance | 1.04 | 0.82, 1.31 | 0.7 | 0.78 | 0.59, 1.03 | 0.08 | 0.68 | 0.54, 0.86 | **0.001** |
| Pathology stage |  |  |  |  |  |  |  |  |  |
| pT0/pTa/pTis/pT1 | Ref | Ref |  | Ref | Ref |  | Ref | Ref |  |
| pT2 | 3.51 | 2.07, 5.93 | **<0.001** | 2.41 | 1.37, 4.25 | **0.002** | 1.65 | 1.07, 2.55 | **0.02** |
| pT3/pT4 | 6.87 | 4.35, 10.9 | **<0.001** | 4.75 | 2.92, 7.73 | **<0.001** | 3.31 | 2.33, 4.70 | **<0.001** |
| Lymph node involvement | 1.40 | 1.10, 1.78 | **0.006** | 2.09 | 1.55, 2.80 | **<0.001** | 1.71 | 1.32, 2.20 | **<0.001** |
| Positive soft tissue surgical margin | 1.47 | 1.10, 1.96 | **0.01** | 1.67 | 1.19, 2.36 | **0.003** | 1.64 | 1.22, 2.20 | **<0.001** |
| NAC | 1.20 | 0.78, 1.85 | 0.4 | 1.12 | 0.65, 1.93 | 0.7 | 0.96 | 0.60, 1.53 | 0.9 |
| CIS = carcinoma in situ; FSA = frozen section analysis; HR = Hazard Ratio; CI = Confidence Interval; NAC = neoadjuvant chemotherapy | | | | | | | | | |

**Supplementary Figure 1. Kaplan-Meier analysis for (A) progression-free survival (PFS), (B) cancer-specific survival (CSS), and (C) overall survival (OS) in 887 patients treated with radical cystectomy for urothelial carcinoma of the bladder, stratified according to urethrectomy performance.**

**Supplementary Figure 2. Kaplan-Meier analysis for (A) progression-free survival (PFS), (B) cancer-specific survival (CSS), and (C) overall survival (OS) in patients treated with radical cystectomy for urothelial carcinoma of the bladder, stratified according to urethral frozen section analysis performance.**

**Supplementary Figure 3. Kaplan-Meier analysis for (A) progression-free survival (PFS), (B) cancer-specific survival (CSS), and (C) overall survival (OS) in high-risk patients treated with radical cystectomy for urothelial carcinoma of the bladder, stratified according to urethral frozen section analysis performance.**
